# A 0.0016 mm^2^ 0.64 nJ Leakage-Based CMOS Temperature Sensor

**DOI:** 10.3390/s130912648

**Published:** 2013-09-18

**Authors:** Pablo Ituero, Marisa López-Vallejo, Carlos López-Barrio

**Affiliations:** Departamento de Ingeniería Electrónica, ETSI Telecomunicación, Universidad Politécnica de Madrid, Avenida Complutense 30, 28040 Madrid, Spain; E-Mails: marisa@die.upm.es (M.L.-V.); barrio@die.upm.es (C.L.-B.)

**Keywords:** temperature sensor, CMOS, ratio-based, leakage, DTM (Dynamic Thermal Management)

## Abstract

This paper presents a CMOS temperature sensor based on the thermal dependencies of the leakage currents targeting the 65 nm node. To compensate for the effect of process fluctuations, the proposed sensor realizes the ratio of two measures of the time it takes a capacitor to discharge through a transistor in the subthreshold regime. Furthermore, a novel charging mechanism for the capacitor is proposed to further increase the robustness against fabrication variability. The sensor, including digitization and interfacing, occupies 0.0016 mm^2^ and has an energy consumption of 47.7–633 pJ per sample. The resolution of the sensor is 0.28 °C, and the 3*σ* inaccuracy over the range 40–110 °C is 1.17 °C.

## 1. Introduction

The same technical advances that have permitted the aggressive technology scaling we have and continue to witness, such as sub-wavelength lithography, have also increased the significance and complexity of process variations. Lithographic uncertainties, dopant variations and well-proximity effects, among others, influence the properties of fabricated chips, making the characterization of integrated circuits a complex process that must necessarily cope with the probabilistic distribution of the parameters instead of a set of fixed corner cases. Nonetheless, from a holistic point of view, system features, such as power consumption, mean time to failure or clock frequency, are very much impacted by these fluctuations, and controlling them supposes a big challenge for current designers. In particular, concerning power issues, increasing power densities worsen the thermal impact on reliability and performance, while decreasing supply voltages worsen leakage currents and noise; leakage power varies exponentially with key process parameters, such as gate length, oxide thickness and threshold voltage.

In this context, temperature is by itself a source of new variations—many aging process are tightly coupled with thermal gradients and stresses—but, also, a victim of process uncertainties. Its dependence on power densities along with its relationship to leakage currents make it especially sensitive to all of these issues. Therefore, the importance of Dynamic Thermal Management (DTM) systems with technology scaling has continued to increase.

However, the design of temperature sensors that fulfill the accuracy requirements imposed by DTM policies comprising a small area and power overhead under this scenario of device uncertainties is not a simple task. Process variations can affect the quality of the reference signal or that of the measurement in such a way that the accuracy of the sensor is completely ruined. Traditional bandgap-based temperature sensors employing analog-to-digital converters (ADCs) have overcome these problems at the cost of an elevated area and power overhead that is not suitable for DTM systems.

In this context new types of temperature sensors specially tailored for DTM have been presented in the last few years. They normally have the common feature of employing a time-to-digital converter (TDC) or a frequency-to-digital converter (FDC) to perform the digitization. Several mechanisms have been proposed to implement the sensing part of these sensors:

- Propagation delay of a chain of inverters or a chain of equal logic elements. Normally, the chain is closed and forms a ring oscillator that provides a thermal-dependent frequency. The most important parameter that affects the delay of these structures is the mobility of the carriers, which decreases with temperature; therefore, the frequency has a negative temperature coefficient. However, the delay is also dependent on *V_DD_* and *V_T_*, which degrades the linearity of the response. In the last few years, there has been a lot of research trying to improve the robustness against process and voltage variations and a myriad of these types of monitors can be found in the literature [1–3].
- Propagation delay of a transmission line. In this case, the analog signal is the temperature varying width of a pulse traveling through a transmission line. Again, the carrier mobility dependence on the temperature is the key factor that will govern the sensor response; however, the reduced reliance on other factors will provide it with an extended linearity [4].
- Propagation delay through on-chip resistors. In this case, the varying pulse width is generated employing the thermal dependencies of an on-chip resistor. These sensors have been employed to monitor the temperature of mobile DRAM (Dynamic Random Access Memory), as described in [5].
- Leakage-based. The generation of the pulse is based upon the thermal dependencies of the leakage currents of a transistor [6] or a diode [7]. Ref [8] proposed a technique that performed the ratio between two measures that effectively reduced the dependency against fabrication uncertainties.

This work develops the ratio-based technique proposed in [8] and introduces the design of a CMOS temperature sensor implemented in a 65 nm technology. The sensor performs the ratio of the two measures of the discharge time of a capacitor driven by the leakage current of an NMOS (Negative channel Metal Oxide Semiconductor) transistor; each measure is taken with a different gate voltage. The 65 nm technology node is subjected to intense process variability; therefore, important care was taken in the implementation of the different parts of the sensor, so that the benefits of the technique were not compromised by the surrounding circuitry. In particular, a new charging scheme for the capacitor is proposed that increases the robustness against process variability. The resulting sensor displays a very interesting compromise between area (0.0016 mm^2^), energy overhead per conversion (48–640 pJ) and accuracy (1.17 °C), which makes it very suitable for DTM purposes.

The structure of the paper is the following. Section 2 describes the sensing mechanism and the analytical equations. Section 3 goes through the most important implementation issues of both the sensing part and the digitization and interface circuitry. The characterization of the sensor and the comparison with previous works in the literature are presented in Section 4. Finally, Section 5 draws some conclusions.

## 2. Analytical Description

This paper presents a novel temperature sensor architecture based on the technique introduced in [8], which relies on the ratio of two leakage-dependent measures, as explained next. The thermal dependency of the leakage current, *I_DS_*, of a MOS transistor operating in the subthreshold region is given by the following expression:

(1) $$I_{DS}(T)=K_{1}T^{2}e^{\frac{1}{T}(\frac{V_{GS}}{nk/q}-K_{2})}(1-e^{-\frac{V_{DS}}{kT/q}})$$

where *T* is the temperature, *V_DS_* is the drain-source voltage, *K*_1_ and *K*_2_ are technology parameters, temperature and *V_GS_*-independent, *kT*/*q* is the thermal voltage, *n* is the transistor subthreshold swing coefficient and *V_GS_* is the gate-source voltage.

**Figure 1.**
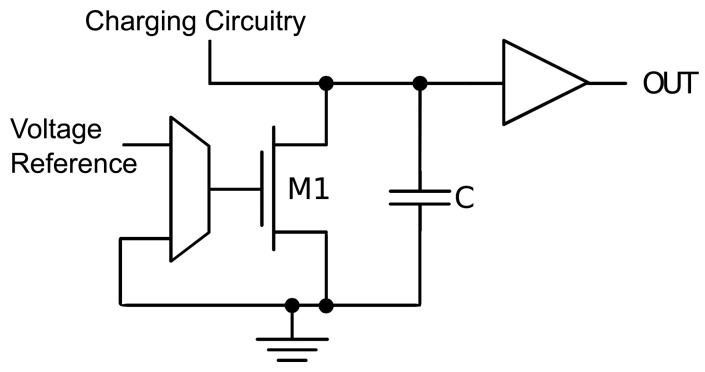
Constituent blocks of the 65 nm thermal sensor.

This expression is influenced by many process-dependent factors, which makes the leakage current one of the most manufacturing-sensitive parameters of a chip. In order to neutralize these dependencies, Ref [8] describes a technique based on the idea that the ratio of two *I_DS_* with identical conditions differentiated just by *V_GS_*—ground in one case and *V_BIAS_* in another— is given by:

(2) $$\frac{K_{1}T^{2}e^{\frac{1}{T}(\frac{V_{\text{BIAS}}}{nk/q}-K_{2})}(1-e^{-\frac{V_{DS}}{kT/q}})}{K_{1}T^{2}e^{\frac{-K_{2}}{T}}(1-e^{-\frac{V_{DS}}{kT/q}})}=e^{\frac{\frac{V_{\text{BIAS}}}{nk/q}}{T}}$$

which effectively eliminates most dependencies on technological parameters affected by variations. In order to exploit this property, the structure of Figure 1 charges the capacitance, *C*, and lets the charge leak away through transistor *M*1, whose *V_GS_* can be set at either ground or *V_BIAS_*. The *OUT* signal corresponds to a pulse, whose width is dependent on the leakage current, *i.e.*, the temperature and the value of *V_GS_*. The following expression gives the discharge time of *C* through the leakage current of *M*1 from *V_DS_* = *V*_1_ to *V_DS_* = *V*_2_:

(3) $$\Delta t_{V_{1}\to V_{2}}\left(T\right)=\frac{C}{K_{1}T^{2}}\frac{kT}{q}{\text{e}}^{\frac{1}{T}(\frac{V_{G}}{nk/q}-K_{2})}[\ln(e^{\frac{V_{1}}{kT/q}}-1)-\ln(e^{\frac{V_{2}}{kT/q}}-1)]$$

which is the duration of a pulse that can be sensed and digitized at *OUT*. Performing the ratio of two measurements, one with *V_G_* = *GND* and another with *V_G_* = *V_BIAS_*, yields:

(4) $$\frac{\Delta t_{V_{1}\to V_{2}}(T)|V_{G}=0}{\Delta t_{V_{1}\to V_{2}}(T)|V_{G}=V_{\text{BIAS}}}=e^{\frac{V_{\text{BIAS}}}{nkT/q}}$$

Following the philosophy presented in [6], carrying this expression into the logarithm domain produces:

(5) $$\log[\frac{\Delta t_{V_{1}\to V_{2}}(T)|V_{GS}=0}{\Delta t_{V_{1}\to V_{2}}(T)|V_{GS}=V_{\text{BIAS}}}]=\frac{V_{\text{BIAS}}}{nkT/q}$$

an expression that does not display any dependency on the size of the capacitor, the voltage the node is charged at, the threshold of the inverter or the device parameters of M1, beyond the subthreshold swing, *n*. Experimental data show that *n* is a function of the channel length and the interface state density [9]. Section 4 will show that its variations across the process spectrum have little impact on the sensor measurement. The parameter that will most affect the behavior of the sensor is *V_BIAS_*, as it is directly proportional to the measurement. The robustness of *V_BIAS_* against process, voltage and temperature (PVT) variations is one of the key factors that will determine the quality of the sensor. As explained in [10], this type of leakage-based sensors displays little dependence on power supply (*V_DD_*) variations; as a matter of fact, in this case, *V_DD_* fluctuations just alter the response of the voltage reference, *i.e.*, the value of *V_BIAS_*, and the dependence of the sensor on *V_DD_* is in this way.

These characteristics make the sensor especially suitable for tracking the temperature in the current environment of exacerbated process variations. Achieving a sensor transfer function that follows the ideal analytical description faithfully requires careful design and implementation strategies. The next section presents the most relevant implementation issues of each constituent block of the sensor.

## 3. Implementation Issues

**Figure 2.**
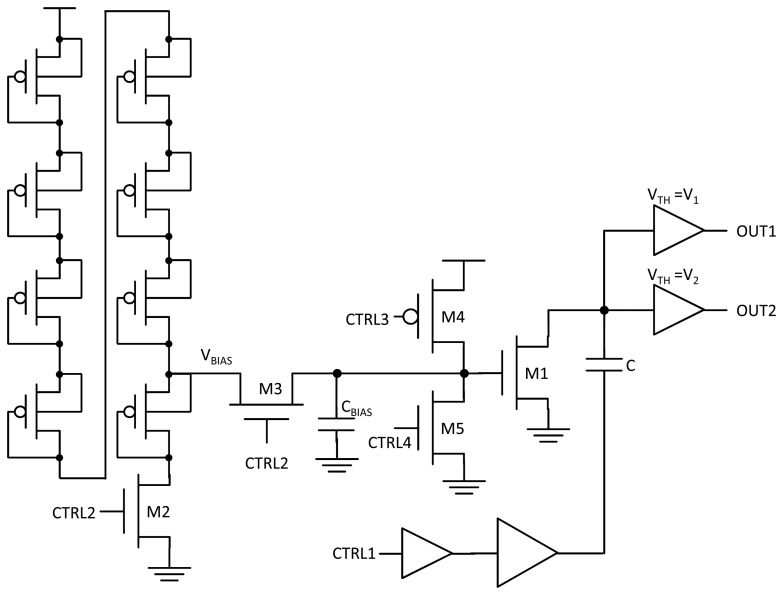
Complete sensing part schematic.

Bearing in mind all the elements previously described, the schematic of the whole sensing part is depicted in Figure 2. As shown, the multiplexer at the gate of transistor M1 is implemented by means of tri-state logic through transistors M3, M4 and M5.

Transistor M1 is the leaking source that controls the behavior of the sensor; the rest of the circuitry is described in the following sections.

### 3.1. Charging Circuitry Implementation Issues

The charging circuitry is one of the elements that has the highest potential to cause errors in the sensors response with respect to the ideal behavior defined by Equation (5). The reason behind this is that this element needs to, somehow, write a value in the measurement node and, then, disconnect it, so that the capacitor gets discharged through M1. However, this disconnection may imply leaving some “off” transistors connected to the floating node and, thus, introducing a certain amount of leakage current, which can seriously interfere with that of M1. Within-die variations of current technologies introduce a high degree of uncontrollability no matter the sizing of the transistors.

**Figure 3.**
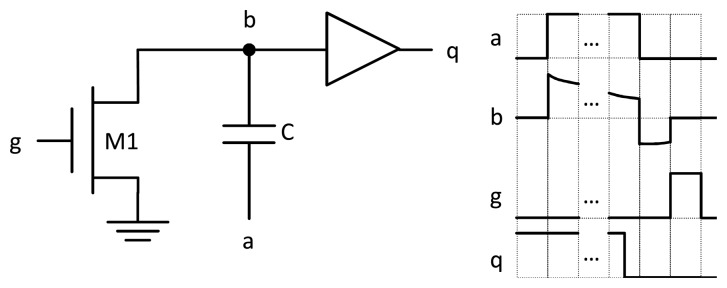
Charging circuitry explanation: On the left, the charge pump example circuit. On the right, transient signals for the charge pump.

In order to overcome this situation, a charge pump was designed to charge the measurement node through capacitance C. According to the charge conservation principle, any instantaneous change in the voltage of a capacitor terminal is followed by the other terminal. In a real circuit, there is no such thing as instantaneous voltage changes; however, strict requirements of the rise and fall times of a signal can be fulfilled by making it pass through a correctly-sized chain of inverters. Employing the circuit depicted in Figure 3, let us explain the charging mechanism. At the departure point, we suppose that capacitor C is discharged and that both nodes, a and b, are set to a voltage equal to ground. Next, if a rising pulse is applied between ground and *V_DD_* at node a, ideally, node b will also be raised to *V_DD_*. Then, the node is discharged through the leakage currents of transistor M1. When the output of the sensor, node q, changes, because the voltage at node b surpasses the threshold of the buffer, *V_inv_*, a falling pulse is applied at node a. Following the charge conservation principle, the voltage across the capacitor remains constant, and the voltage at node b will be now set at approximately–*V_inv_*. In order to return to the initial state, transistor M1 is set temporarily to an on state, which takes node b to a ground voltage.

Since the value at which the node is charged is variable due to the process fluctuations, clock skew and jitter, an extra buffer was introduced at the output of the sensor with a threshold above that of the first buffer, as shown in Figure 2. In this way, there is an extended control over the measurement interval, since both *V*_1_ and *V*_2_ are fixed by the buffers.

### 3.2. Bias Voltage Implementation Issues

As shown in Equation (5), the characteristics of the voltage reference are a key factor for the transfer function of the sensor. A voltage divider composed of a set of equal resistances was chosen as the best option to provide the reference subthreshold voltage, because it supposes a good compromise between power and area overheads and robustness against environmental fluctuations. PMOS (Positive channel Metal Oxide Semiconductor) transistors are employed, because using an n-well technology allows the modification of their substrate voltage. This is an important point, because body effect-induced *V_TH_* alterations can vary with technology corners.

This structure is not affected by temperature changes and is very insensitive to process and mismatch variations. However, the proposed divider displays a linear dependency on *V_DD_*, which imposes very strict restrictions on the *V_DD_* power network. Furthermore, there is a trade-off between the output resistance of the voltage reference and the static power consumption. In order to provide the voltage reference with a mechanism to disconnect when not in use, certain control circuitry must be added, as shown in Figure 2.

The multiplexer at the gate of transistor M1 is implemented by means of tri-state logic through transistors M3, M4 and M5. When M3 is on, the gate is driven with the bias voltage; when M5 is active, the gate is grounded; and when M4 is on, the gate is set to *V_DD_*, as required by the charging circuitry. Their corresponding control signals are asserted by a finite state machine.

### 3.3. V_BIAS_ Stability Issues

Charge conservation effects can distort the quality of *V_BIAS_*. Specifically, charge conservation of the parasitic capacitor between the gate and the drain of transistor M1 makes the voltage of the gate of the transistor tend to follow that of the drain of the transistor. This implies a fall in *V_BIAS_* when capacitor C is getting discharged, which translates into unexpected sensor response. The problem is worsened when dealing with relatively high-speed discharge times produced at high temperatures and fast technology corners.

**Figure 4.**
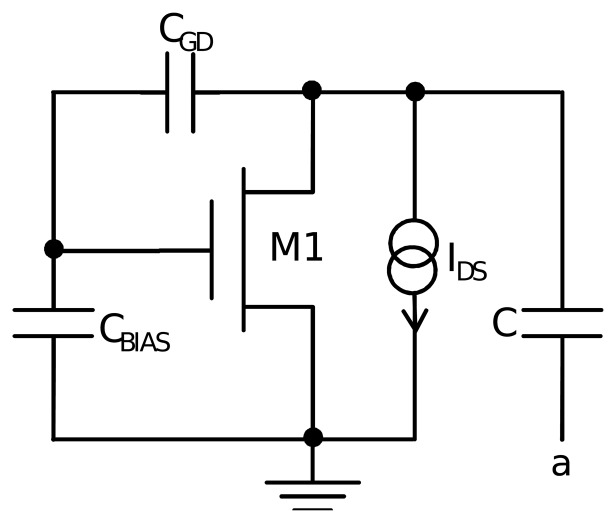
Relevant circuital elements to analyze *V_BIAS_* stability issues.

Figure 4 includes the relevant elements that take part in the mechanism. In the center, we have transistor M1. Capacitor C is between the drain and ground when a = GND. There is a parasitic capacitor in between the drain and the gate, *C_GD_*. Furthermore, there is another parasitic capacitor between the gate and ground, *C_BIAS_*. Finally, the current drawn by the drain is modeled as a current source from the drain to the ground. Supposing that the current source uniquely extracts charge from C and that this produces a voltage swing at the drain from initial *V_Di_* to final *V_Df_*, the following expressions define the behavior of the charges and voltages of *C_GD_* and *C_BIAS_*:

(6) $$(V_{Di}-V_{Gi})C_{GD}+V_{Gi}C_{\text{BIAS}}=(V_{Df}-V_{Gf})C_{GD}+C_{GD}+V_{Gf}C_{\text{BIAS}}$$

which yields:

(7) $$\Delta V_{G}=V_{Gi}-V_{Gf}=\frac{C_{GD}}{C_{\text{BIAS}}-C_{GD}}(V_{Di}-V_{Df})$$

From this equation, it can be seen that, actually, *C_BIAS_* is a design parameter that can be controlled in order to increment the stability of *V_BIAS_*. From a simplistic analysis, increasing *C_BIAS_* indefinitely could solve this problem; however, such an action, apart from ruining the area budget, would require a voltage reference able to charge this huge capacitor in short time, *i.e.*, with an extremely low output resistance. The voltage reference is made of a set PMOS transistors forced to work in saturation, and the only way to increase the current they can provide or reduce the output resistance of the structure is to increase the widths of the transistors. This, in turn, increases the area and the static power consumption when the generator is activated. Furthermore, the size of C has a certain impact on all these matters, because it is directly proportional to the speed of the voltage swing at the drain, and the same applies to the size of transistor M1 itself. All the previous considerations must be taken into account when adjusting the design parameters in order to reach a convenient trade-off between the *V_BIAS_* fluctuation tolerance, the area and power budgets.

### 3.4. Digitization and Interfacing

As far as the digitization of the sensor is concerned, the fundamental principle that is employed is based upon the ideas presented in [6], where it was proposed to perform a logarithmic time-to-digital conversion by means of a logarithmic counter. The logarithmic counter is composed of two counters, a tree of frequency dividers and an LUT. One counter stores the integer part of the logarithmic count, and the other one stores the fractional part in the linear domain. The frequency divider controls the increment rate of the first counter, and the LUT transforms the content of the second counter into the logarithmic domain. In the case of this work, it was necessary to adapt the design to the peculiarities of having two different measures over different interval ranges.

Each of the two measures presents different characteristics in terms of the range of pulse durations (considering all kinds of PVT variations) and maximum error with respect to an ideal linear behavior. The most restrictive count, which is the shortest (*V_G_* = *V_BIAS_*), sets the sizing requirements of the internal counters of the logarithmic counter. The effect on the other measurement is an oversized resolution that is beyond the actual precision of the measure. In any case, the outgoing frequency of the divider is configurable; therefore, each counting process can have a different base frequency.

**Figure 5.**
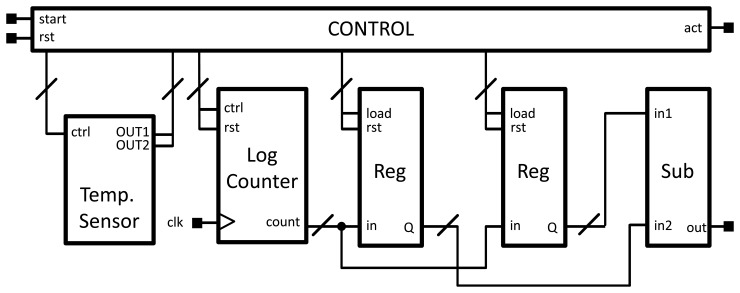
Constituent blocks of the 65 nm thermal sensor.

Figure 5 shows the sensor along with the interfacing blocks. When a start signal is received, the count, related to the first measurement with *V_G_* = *V_BIAS_*, starts. When it finishes, the result is stored in the first register, and the second count, with *V_G_* = *GND* begins. When the second count is over, the result is stored in the second register, and the act signal is activated, meaning that the subtractor yields a valid output. A small controller is in charge of the external protocol and produces the required control signals for each block.

Considering the whole system, the fact that two measurements are subtracted implies that the errors carried along with each measure—due to the inaccuracies of the model or quantization noise—potentially accumulate in the subtraction. In order to equalize the actual precision of the sensor with its resolution, or the number of bits it yields, the LUT of the logarithmic counter must be sized in accordance with the accumulated error.

## 4. Characterization

The sensor has been designed and laid out using a 9-metal 65 nm CMOS technology from TSMC™ powered at 1 V. The experimental work, simulations and layouts have been carried out in the Cadence™ environment. All data come from post-place-and-route simulations.

**Figure 6.**
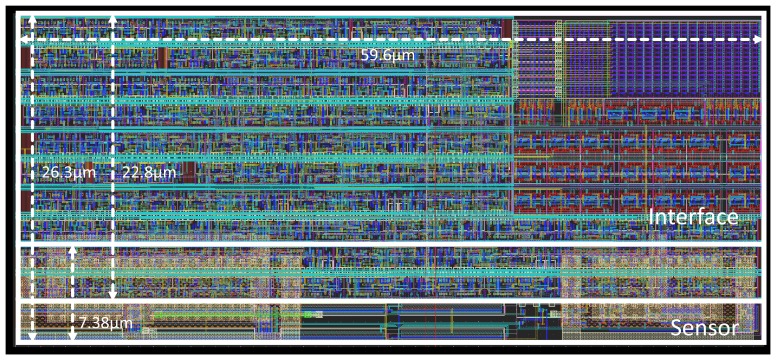
Layout of the 65 nm temperature sensor.

Figure 6 shows the layout of the complete sensor comprising the sensing and the digitization part. Capacitors *C* and *C_BIAS_* (120 fF and 200 fF, respectively) entail the most restrictive individual elements of the sensor in terms of area due to both the actual area physical requirements to construct the capacitance and the separation constraints imposed by the technology. They are implemented using MIMcapacitors between metal-8 and metal-9 layers. This type of capacitor allows the introduction of logic underneath it, permitting the overlap of the sensing and digitization parts. As shown in Table 1, the sensing part takes 7.38 μm × 59.6 μm, 439.8 μm^2^ and 28.1% of the total, the digitization part takes 22.8 μm × 59.6 μm, 1,358.9 μm^2^ and 86.6% of the total. The complete sensor takes 26.3 μm × 59.6 μm, 1,567.5 μm^2^. The voltage reference was designed to provide a voltage of 0.125 V.

### 4.1. Sensitivity and Resolution under Nominal Conditions

As expressed by Equation (5), the sensor presents a transfer function that is directly proportional to the inverse of the temperature. For a limited range of temperatures, this function can be approximated by a linear response, albeit a certain degree of curvature will always be present. Furthermore, second and greater order effects not taken into account by our model would introduce a certain distortion. These limitations will be responsible for the inaccuracy of the sensor under nominal conditions. In order to provide a better approximation for the linear response and considering the field of application for the monitor—on-chip thermal management—we restrict the range of temperatures to 40–110 °C.

As far as the power-supply sensitivity of the sensor is concerned, it is very difficult to establish a closed form expression of the degradation caused by fast transient *V_DD_* droops, the most probable type of power noise. However, it is possible to analyze the pessimistic case when throughout the measurement, *V_DD_* remains at a constant value. From Equation (5), the predicted temperature, *T_p_*, as a function of the output code, *O_code_*, is given by

(8) $$T_{p}=\frac{V_{\text{BIAS}}}{nkO_{\text{code}}/q}$$

considering that *V_BIAS_* = *V_DD_*/8, the sensitivity of the sensor to *V_DD_* is given by

(9) $$\frac{\partial T_{p}}{\partial V_{dd}}=\frac{1}{8nkO_{\text{code}}/q}$$

which varies with the output code along the temperature range. For the implementation presented in this work and for the range of temperatures, the sensor displays a sensitivity on *V_DD_* of 0.21–0.26 °C/mV.

**Figure 7.**
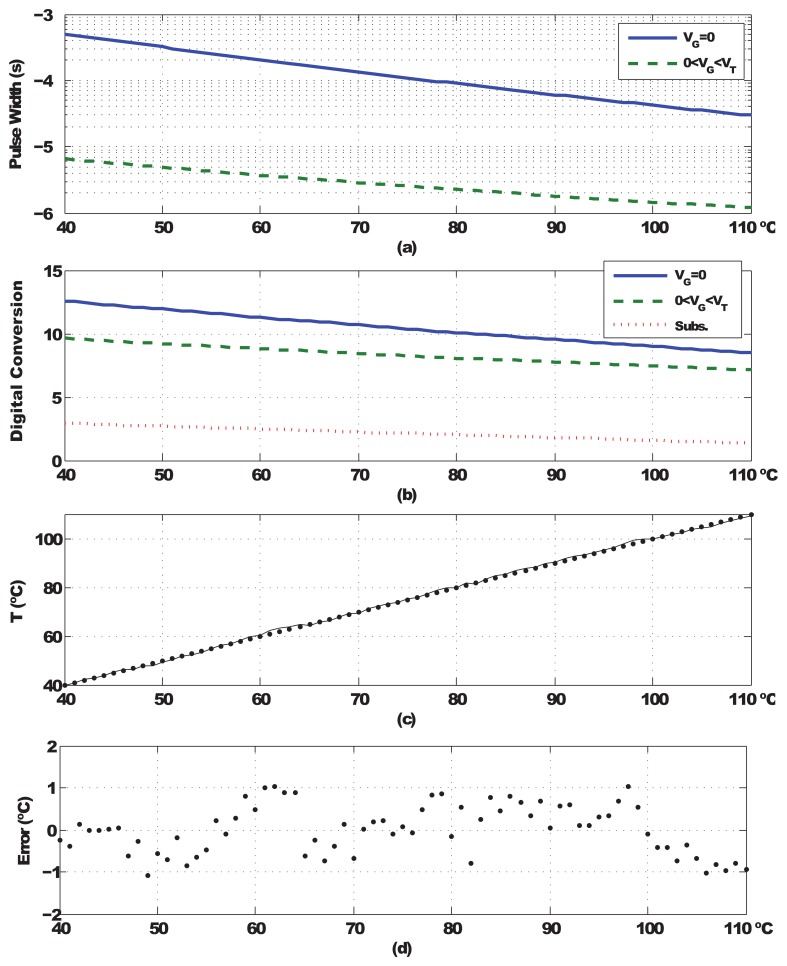
Characterization of the sensor under nominal conditions.

Figure 7a–d shows the results of the sensor characterization performing post-place-and-route simulations and taking one sample for each integer in the range. The variation of the pulse widths on the temperature is displayed in Figure 7a, as shown; the first measure realized putting the gate voltage of transistor M1 at GND goes from 477 μs at 40 °C to 6.33 μs at 110 °C; the second, realized setting M1's *V_G_* =*V_BIAS_*, varies from 28.7 μs at 40 °C to 1.17 μs at 110 °C. Figure 7b shows the digital signal output by the logarithmic counter for each of the measures (*V_G_* = *GND*, solid, *V_G_* = *V_BIAS_*, dashed) along with the result of the subtraction (dotted). Figure 7c displays the characteristic curve of the sensor under nominal conditions. The deviation from the linear regression of all points provides the error committed in each measurement. Figure 7d shows the error for each temperature; the 3*σ* value of the error under these conditions is 0.96 °C. Assuming that the outputs of the sensor at 40 °C and 110 °C are X and Y, respectively, then the effective resolution can be calculated as (110 – 40)/(*Y* – *X*). Under nominal conditions, the sensor displays an effective resolution of ±0.20 °C. Note that these figures do not include the effect of the thermal noise present at the floating capacitor; in the worst case, for the maximum temperature, 110 °C, the root mean square (RMS) value of this noise is approximately 210 μV, which has a negligible impact compared to the non-linearities of the sensor response.

### 4.2. Power and Energy under Nominal Conditions

**Figure 8.**
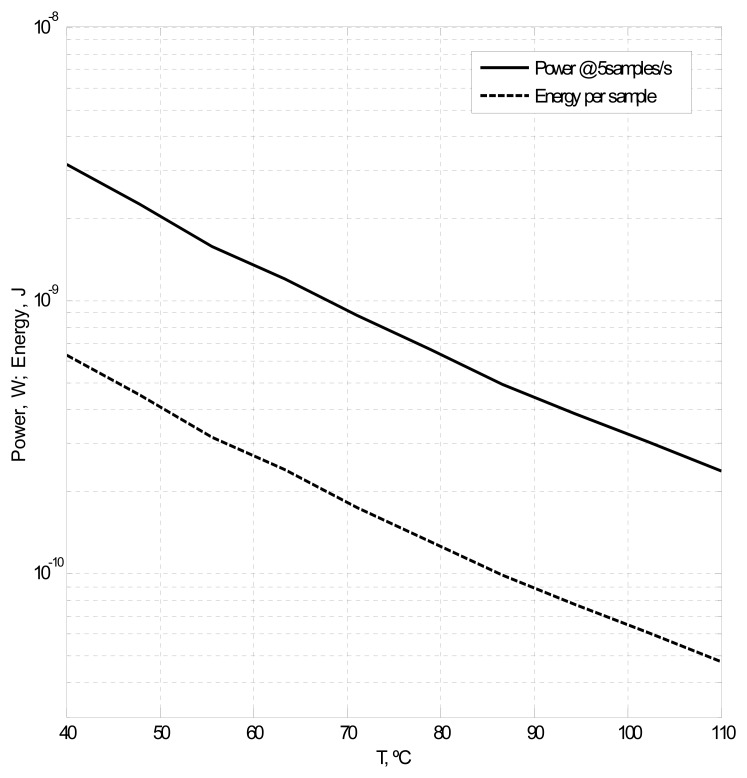
Power consumption at five samples per second and the energy per sample of the sensor under nominal conditions.

Regarding the power consumption and the energy per reading, they depend on the temperature, as the length of the pulses varies and, thus, the logarithmic counter is active for different times. The power consumed by the sensing part is negligible when compared to the digitization part; one might think that the voltage divider—formed by a chain of PMOS devices connecting *V_DD_* and GND—could represent a significant contribution to the power of the whole system; however, this divider is only active during the duration of the second measure and, in any case, is very small compared to the digitization circuitry. Figure 8 displays the dependence of the power consumption at five samples per second and the energy per sample on the temperature. The power has a variation from 3.16 to 0.239 nW (40–110 °C), and the energy ranges from 633 to 47.7 pJ for the same temperature range.

### 4.3. Effects of the Process Variability

Fluctuations in *V_BIAS_* along with process dependencies of the second order and beyond effects not taken into account by the analytical model will impact the accuracy of the sensor.

In order to estimate the effects of fabrication uncertainties on our sensor, we have performed 100 Monte Carlo simulations considering process variations and mismatches. The vendor provides a description of the probability distribution for each parameter of the transistor model. The electronic design automation (EDA) tool produces a cloud of values for each parameter accordingly to their probability distributions, so that a set of 100 test circuits—representing different technology corners—are produced. For each integer temperature in the interval, a transient simulation is performed for each test circuit, so that, as a whole, 7000 simulations are performed. The resulting data will ideally display the same probability characteristics as a set of fabricated circuits.

**Figure 9.**
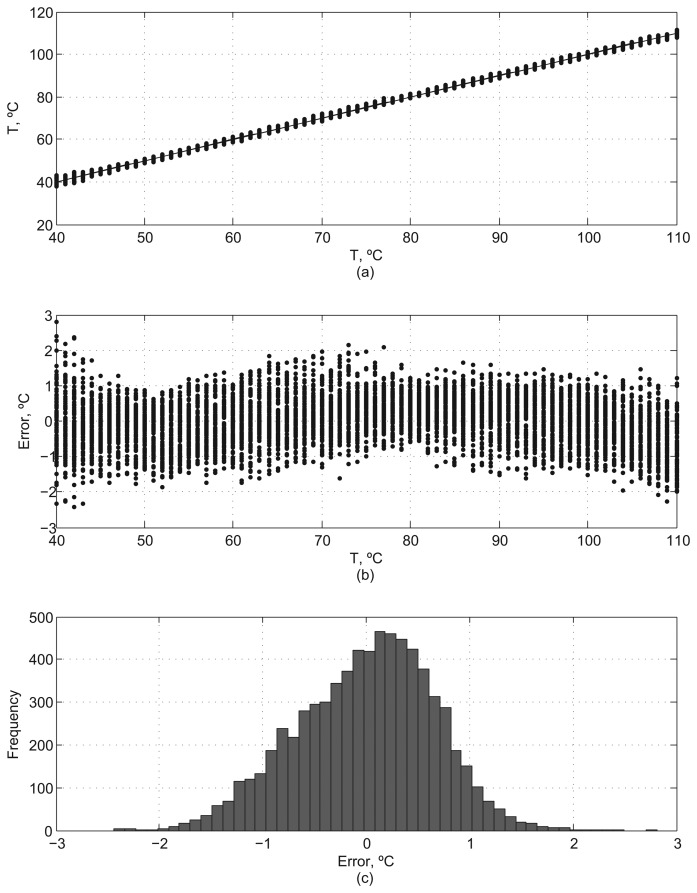
Sensor characterization under the effects of process variability.

Figure 9a–c shows the results of the sensor characterization considering process variability. Figures 9a,b are the equivalent to Figures 7c–d, considering the probability distributions after calibration. Figure 9c displays the distribution of errors for the 7000 samples, which presents a 3*σ* value of 1.18 °C.

### 4.4. Comparison with Previous Works

In order to establish a comparison of the presented sensor to those in the state-of-the-art, we have accessed the excellent on-line temperature sensor survey provided by K.A.A. Makinwa [11], who unselfishly keeps an up-to-date Excel file, including data published over the last 25 years. Among the numerous works that compose the survey, we have selected those sensors that are most suitable to DTM purposes, due to their reduced area and energy consumption, along with an acceptable accuracy. Ref [1,3,7] are sensors that achieved extremely reduced areas for different technology generations; the sensors in [2,12], resistor- and MOS-based, respectively, achieve very low energy per conversion in the 180 nm node; Ref [13] supposes a very good compromise between area and energy for a 65 nm technology.

As shown in Table 2, the present work is very competitive in terms of area, energy consumption and accuracy. The smallness and low power consumption of the sensor are very interesting features for DTM purposes. First, the sensor is highly insensitive to spatial thermal gradients, because of the reduced area of the sensing part. Second, the low power dissipation makes the system very robust against self-heating issues.

## 5. Conclusions

In this paper, we have presented a temperature sensor in the 65 nm technology node hardened against process variations, based on the technique presented in [8]. Departing from a charged floating node, the sensor measures the discharge time, first, through a transistor with *V_G_* = *GND* and, second, through a transistor with 0 < *V_G_*< *V_T_*. The ratio between these two measures in the logarithmic domain has proven to have a convenient dependency on the temperature, while displaying an important robustness against process variations. Several implementation issues have been addressed, including the utilization of a charge pump to charge the floating node.

The sensor occupies an area of 0.0016 mm^2^. The energy per conversion is 48–640 pJ, and the 3*σ* inaccuracy is 1.17 °C. These characteristics fall within the acceptable range for DTM policies and are very attractive compared to previous works. Furthermore, the sensing part of our proposal is so small and easy to implement, that it could possibly be included in any standard-cell library, leaving the interface up to the designer's necessities.

## Figures and Tables

**Table 1.** Area characterization of the 65 nm sensor.

	**Dimensions (μm)**	**Area (μm^2^)**	**Percentage (%)**
Sensing Part	7.38 × 59.6	439.8	28.1
Digitization Part	22.8 × 59.6	1,358.9	86.6
Complete Sensor	26.3 × 59.6	1,567.5	100

**Table 2.** Previous works comparison.

**Source**	**Year**	**Technology [nm]**	**Area [mm^2^]**	**Error [°C]**	**Minimum [°C]**	**Maximum [°C]**	**nJ**
Proposed	2013	65	0.0016	1.17	40	110	0.64
[7]	2012	32	0.001	5.2	0	100	40
[1]	2009	65	0.0066	5.6	–40	110	1.1
[3]	2010	90	0.00375	1.2	20	130	91
[13]	2013	65	0.008	3.0	0	110	1.1
[12]	2011	180	0.18	1	0	100	0.3
[2]	2009	180	0.032	1.8	0	100	0.41
